# Effects of repeated transcranial direct current stimulation on smoking, craving and brain reactivity to smoking cues

**DOI:** 10.1038/s41598-018-27057-1

**Published:** 2018-06-07

**Authors:** Marine Mondino, David Luck, Stéphanie Grot, Dominique Januel, Marie-Françoise Suaud-Chagny, Emmanuel Poulet, Jérôme Brunelin

**Affiliations:** 1 INSERM, U1028; CNRS, UMR5292; Lyon Neuroscience Research Center, Psychiatric Disorders: from Resistance to Response Team, Lyon, F-69000 France; 2 University Lyon 1, Villeurbanne, F-69000 France; 3 Centre Hospitalier Le Vinatier, Bron, France; 4 Department of psychiatry, Faculty of Medicine, Université de Montréal, Montreal, Canada; 5 Centre de recherche, Institut universitaire en santé mentale de Montréal, Montreal, Canada; 6 URC, Pole G03, EPS Ville Evrard, 93300 Neuilly Sur Marne, France

## Abstract

Recent studies have shown that transcranial direct current stimulation (tDCS) may reduce craving and smoking. However, little is known regarding brain correlates of these behavioral changes. We aimed to evaluate whether 10 sessions of tDCS modulate cigarette consumption, craving and brain reactivity to smoking cues in subjects with tobacco use disorder (TUD). In a double blind parallel-arms study, 29 subjects with TUD who wished to quit smoking were randomly assigned to receive 10 sessions of either active or sham tDCS applied with the anode over the right dorsolateral prefrontal cortex (DLPFC) and a large cathode over the left occipital region. As compared to sham, active tDCS significantly reduced smoking craving and increased brain reactivity to smoking-cues within the right posterior cingulate, as measured with a functional magnetic resonance imaging event-related paradigm. However, we failed to find a significant difference between active and sham groups regarding the self-reported number of cigarettes smoked and the exhaled carbon monoxide during one month. These findings suggested that 10 sessions of tDCS over the right DLPFC may reduce craving by modulating activity within the resisting-to-smoke network but might not be significantly more effective than sham to decrease cigarette consumption.

## Introduction

Smoking is the leading preventable cause of disease and premature death in the world. Several strategies are used to prevent and control tobacco consumption over the world such as prevention campaigns and smoke-free legislations. However, still 21% of the worldwide population were current smokers in 2013^1^. Among them, 70% want to quit smoking and 40% try to quit each year. Smoking cessation is not easy as tobacco dependence is a cluster of behavioral, cognitive and physiological phenomena. Current available approaches for smoking cessation include cognitive-behavioral therapies, nicotine-replacement therapies, pharmaceutical treatments such as bupropion and varenicline^2^ and combination of these techniques^3^. More precisely, a meta-analysis of pharmacological interventions for smoking cessation have reported that both nicotine-replacement therapies and bupropion are similarly superior to placebo in helping people quit smoking (odd-ratios -OR- of 1.84 (with 95% credible interval -CI −1.71 to 1.99) and 1.82 (95% CI 1.60 to 2.06) respectively) and that varenicline is more effective than single forms of both nicotine-replacement therapies and bupropion, when compared with placebo (OR 2.88; 95% CI 2.40 to 3.47)^2^. However, despite these existing approaches, most quit attempts fail, and relapse to smoking is common^4^. New alternatives are thus needed to help smokers who wish to quit smoking.

Among the new alternatives proposed in smoking cessation, noninvasive brain stimulation techniques such as transcranial direct current stimulation (tDCS) seem promising. tDCS consists in applying a low intensity current through the scalp using two electrodes. It allows modulation of brain activity and connectivity noninvasively in living humans^5^. tDCS has been shown to modulate behaviors and reduce symptoms in several psychiatric conditions^6^, often by targeting the dorsolateral prefrontal cortex (DLPFC). The DLPFC is involved in tobacco use disorder (TUD) together with a complex brain network including subcortical areas such as the amygdala and cortical areas such as the medial prefrontal cortex^7^. More precisely, the DLPFC is part of the brain network involved in smoking cue-reactivity^8^. The DLPFC is also involved in risk taking and decision making processes, processes that are impaired in subjects with TUD^9^. Thus, the DLPFC seems to play a key role in TUD and to be a good target to modulate with tDCS.

Several studies have investigated the effects of tDCS in subjects with TUD and found that tDCS can reduce craving^10,11^ and smoking^12,13^ when applied over either the left or right DLPFC and that repeated sessions of tDCS can have a cumulative effect on smoking behavior^11^. More precisely, the first published study showed that one session of tDCS applied either with the anode over the left DLPFC and the cathode over the right DLPFC or with the anode over the right DLPFC and the cathode over the left DLPFC can reduce cue-induced craving^10^. Two subsequent studies have investigated the effect of repeated sessions of tDCS on cue-induced craving and smoking. In the first one, Boggio *et al*.^11^ showed a cumulative effect of 5 tDCS sessions applied with the anode over the left DLPFC and the cathode over the right DLPFC on reducing cue-induced craving and also reported a 30% decrease in the number of cigarettes smoked. In a preliminary study, Fecteau *et al*.^13^ reported that 5 sessions of tDCS applied with the anode over the right DLPFC and the cathode over the left DLPFC reduce the desire to smoke and the number of cigarettes smoked for at least 4 days after the end of the tDCS regimen. However, some negative findings on craving^14^ and smoking^12^ have also been reported. Namely, Xu *et al*.^14^ failed to find a significant effect of a single session of tDCS applied with the anode placed over the left DLPFC and the cathode over the right supraorbital region on craving after overnight abstinence. Falcone *et al*.^12^ found a significant increase in latency to smoke and a significant decrease in the total number of cigarettes smoked in one hour following a single tDCS session applied with the same electrode montage but no effect on self-reported number of cigarettes smoked in the 24 following hours. One hypothesis that can explain these mixed findings is the number of tDCS sessions. Indeed, in recent clinical trials showing therapeutic effects of tDCS, at least 10 repeated sessions were delivered^6^. We thus believe that there is a crucial need for studies investigating the effect of repeated sessions of tDCS on smoking behavior. In addition, most of the previous studies suffered from a lack of an objective measure of smoking behavior, few investigations of the duration of the reported effect (the longest follow-up period being 4 days^13^) and no investigation of the brain correlates of the behavioral changes. In order to address these limitations, we designed a study including an objective measure of smoking, a better investigation of the duration of the effects and their brain correlates.

In the present study, we aimed to investigate the effects of repeated sessions of tDCS on smoking, craving and brain reactivity to smoking cues. We hypothesized that 10 sessions of tDCS applied over the right DLPFC will decrease smoking and craving in subjects who wish to quit. This hypothesis is based on results from a meta-analysis showing that despite the difference did not reach significance, noninvasive brain stimulation may have greater effect on craving when targeting the right DLPFC as compared to the left DLPFC^15^. We chose a 10-session regimen in line with our previous studies^6^. We investigated the duration of the effects with a one-month follow-up and the effects on brain reactivity to smoking cues using functional magnetic resonance imaging (fMRI) before and after the 10 sessions of tDCS. Reactivity to smoking cues has been associated with relapse and its brain correlates has been largely studied using fMRI^8^. Namely, meta-analyses of smoking cue-reactivity fMRI studies highlighted that smoking cues evoked increased BOLD activity within the dorsal and medial prefrontal cortex, the anterior cingulate, the insula, the dorsal striatum, the posterior cingulate, the precuneus and the extended visual system^8,16^. It seems thus relevant to investigate whether tDCS modulates this brain reactivity using fMRI. We hypothesized that active tDCS will reduce brain reactivity to smoking cues in these structures and especially in the prefrontal structures (DLPFC and medial prefrontal cortex), given that the DLPFC was targeted with anodal-tDCS.

## Methods

### Study design

The study was reported according to the CONSORT (Consolidated Standards of Reporting Trials) guidelines (see Supplementary Material S2 for CONSORT flowchart). The study used a double blind randomized parallel-arms design in which participants received 10 sessions (2 sessions per day for 5 consecutive days) of either active or sham tDCS over the right DLPFC. Time course of the study is depicted in Supplementary Fig. S1. Participants were invited to take part in 7 visits (V0–V6) at the laboratory over the course of one month: the inclusion visit (V0), the five consecutive days of tDCS (V1 to V5) and a visit at one-month follow-up (V6). V1 took place on the Monday following the inclusion visit (V0) and included the 1^st^ fMRI scan followed immediately by the first tDCS session and a second tDCS session at least 2 hours after. V2 to V4 (from Tuesday to Thursday) included 2 tDCS sessions per day separated by at least 2 hours. V5 (on Friday) included 2 tDCS sessions separated by at least 2 hours and the 2^nd^ fMRI scan immediately after the last tDCS session. V6 took place 28 days after V1. Participants were asked to fill out a daily cigarette diary during one month and levels of carbon monoxide (CO) in exhaled breath were measured before the first (V1), after the last tDCS session (V5) and at one-month follow-up (V6). Participants performed a cue-reactivity task during fMRI scanning immediately before the first and after the last tDCS session. Smoking craving was measured before and after each tDCS session.

### Ethics statement

The study was approved by a local ethical committee (*CPP Sud*-*Est III*), and registered in ClinicalTrials.gov database (NCT01288183; First Posted: 02/02/2011; see https://clinicaltrials.gov/ct2/show/NCT01288183 for protocol details). All methods were performed in accordance with the relevant guidelines and regulations. All participants provided written informed consent after a full description of the study.

### Participants

Sample size was estimated Using G * Power. We calculated that a sample of 34 participants would be required to detect a medium effect (f = 0.25) with 80% power and α = 0.05. The effect size of f = 0.25 was calculated based on Fregni *et al*.’s study^10^ and corresponds to the effect sizes reported by a meta-analysis estimating the effects of tDCS on craving^15^. We estimated a 10% attrition rate and thus planned to recruit 38 participants. Participants were recruited via advertisements posted on bulletin boards and emails sent to the student’s mailing list of the University of Lyon between April 2011 and April 2014. Thirty-eight adults with TUD were enrolled in the study after completing an in-person screening interview (Supplementary Fig. S2). Among them, 9 dropped out of the study after completing the inclusion visit (V0) and before V1 (attrition rate: 24%). Reasons for dropping out were: schedule issues (n = 2), reported vertigo on the morning of the first day of treatment before tDCS application (n = 1), did not come at the first tDCS day without giving reasons (n = 6). Twenty-nine participants were thus considered for analysis. All participants were 18–55 years old, smoked between 10 and 25 cigarettes per day, reported a moderate to severe nicotine dependence level (score ≥5 at the Fagerström Test for Nicotine Dependence^17^) and wished to quit smoking. Individuals were excluded if they were taking psychotropic medications, had psychiatric disorders other than TUD, had used medications for smoking-cessation during the previous year, or had any contraindication to MRI. Participants were assessed on mood (Beck Depression Inventory^18^) and motivation to quit smoking (Q-MAT^19^). Participants were required not to use nicotine-replacement strategies or medications for smoking-cessation during the protocol time course.

### Blinding strategy

Neither the experimenter nor the participants were aware of the stimulation condition. To do so, the ‘study mode’ of the tDCS stimulator was used: the experimenter entered a pre-programmed code that delivered either active or sham tDCS but was unaware to which condition the codes apply. The list of codes was established by a researcher not involved in tDCS delivery, data collection and analyses. Stimulation condition was randomized using the block method (block size: 4). Blinding integrity was assessed at one-month follow-up by asking participants to guess which tDCS condition they received (3 choices: ‘active’, ‘sham’, ‘do not know’).

### Transcranial Direct Current Stimulation

tDCS was carried out with an Eldith DC stimulator (NeuroConn, GmbH, Germany) using two saline-soaked sponge electrodes applied over the subject’s scalp. Electrodes were placed according to the international 10–20 EEG system. The anode (7 × 5 cm) was positioned midway between F4 and Fp2 to target the right DLPFC. The cathode (10 × 10 cm) was placed over the left occipital region, midway between O1 and T5. A large cathode was used to minimize neuromodulatory effects under the cathode. Indeed, enhancing the size of the electrode reduces the current density under the electrode while keeping the current strength constant^20^. Active tDCS consisted in delivering a constant current of 2 mA for 20 minutes (ramp-up/down: 30 seconds). For the sham tDCS, the 2 mA current was delivered only during the first 40 seconds of the 20-minute stimulation period mimicking somatosensory artifact of active tDCS. Each participant received a total of 10 sessions on 5 consecutive days (2 sessions per day separated by at least 2 hours). Participants were instructed not to smoke during the 90 minutes prior to each tDCS session.

A simulation of the electric field distribution in the brain for the electrode montage used in the experimental protocol was performed using SimNIBS 2.0.1^21^ and  using the standard head model provided by the software (Supplementary Fig. S3). A current intensity of 2 mA was used in the simulation. The mesh and electric field visualization were performed through Gmsh^22^.

### Smoking intake

Primary outcome was self-reported smoking intake assessed using a daily diary in which subjects had to indicate the number of cigarettes smoked each day. They had to start reporting their cigarette consumption three days before their first tDCS session and during 28 days. Levels of exhaled CO were also measured as a measure of smoking intake before the first tDCS session (V1), after the last tDCS session (V5) and at one-month follow-up (V6) using a CO monitor (MicroCo, Milford, MA, USA).

### Smoking craving

Smoking craving was measured before and after each tDCS session using a 5-item Likert-type scale questionnaire of smoking urge based on Shiffman *et al*.^23^. Each item was rated on a scale ranging from 0 (strongly disagree) to 10 (completely agree). The 5 items were: “I have a desire for a cigarette right now”, “If it was possible, I would smoke right now”, “All I want right now is a cigarette”, “I have an urge for a cigarette”, “I can no longer restrain my desire to smoke”. Responses were averaged over the 5 items to produce a total craving score at each time point.

### fMRI procedure

All participants underwent two fMRI sessions: one immediately before the first tDCS session and one immediately after the end of the 10^th^ tDCS session. During each scan, participants passively viewed 40 smoking, 40 neutral and 10 target images divided into 6 runs of 15 images with approximately half being smoking images, half neutral images and 1–2 targets. Each run lasted between 4.5 and 5 min. Each participant was presented with the same images at each session, however, the order of presentation varied across sessions, both between and within runs. Smoking and neutral images were selected from the International Smoking Image Series, version 1.2^24^. Smoking images included smoking-related content such as people smoking or holding cigarettes, or cigarettes alone. Neutral images were matched for content and involved peoples, hands or objects such as pens. Target images were pictures of animals. To ensure that participants were awake and maintained attention, participants were asked to press a button whenever they saw a target. Each image was presented for 4 seconds separated by a jittered white fixation cross centered on a black screen shown for 15 +/− 5 seconds. Images were presented in a pseudorandom order, with no more than two of the same images type appearing consecutively. All images were projected onto a flat screen positioned behind the participant’s head, which the participant viewed via a mirror mounted on the head coil.

### fMRI acquisition

Images were acquired at the “CERMEP-Imagerie du vivant” imaging center of Lyon (France) on a 1.5T Siemens Sonata scanner with a standard 8-channel head coil. For fMRI, echo planar T2*-weighted axial images were acquired with the following parameters: 26 interleaved slices; repetition time = 2500 ms; echo time = 60 ms; field of view = 240 mm^2^; flip angle = 90°; matrix = 64 × 64 (voxel size: 3.75 × 3.75 × 4 mm). Manual shimming was performed on the whole brain to improve the local field homogeneity and minimize susceptibility artifacts. Before the first fMRI session, a 3D T1-weighted anatomical scan was acquired (176 axial slices; repetition time = 1970 ms; echo time = 3.93 ms; field of view = 256 mm^2^; voxel size = 1 mm^3^).

### Statistical analysis

The main outcome was the number of cigarettes smoked per day. Change in the number of cigarettes smoked over time was analyzed with a linear mixed-effects model, which account for non-independence of observations inherent in repeated-measures data. The model was implemented using the nlme package for linear mixed modeling (lme function) in R software (v.3.4.3)^25^ and included two distinct time periods: during tDCS treatment (Time 1) and the follow-up period (Time 2). The model included fixed effects of Time 1 (linear and quadratic coefficients), Time 2 (linear and quadratic), Group and interactions between Group and all time terms. The intercept and time terms (Time 1 and Time 2) were entered as random effects. An exponential spatial correlation structure for the residuals was considered.

Secondary outcomes were analyzed with SPSS-22 (Statistical Package for the Social Sciences). CO levels in exhaled breath were entered into a repeated-measure ANOVA with stimulation Group (active, sham) as between-factor and Time (V1, V5 and V6) as within-factor.

Changes in scores at the smoking craving questionnaire from pre to post tDCS session (Post-Pre delta) were submitted to a repeated-measure ANOVA with stimulation Group (active, sham) as between-factor and Session (session 1 to session 10) as within-factor.

Characteristics at baseline between the two groups were compared using independent Student’s t tests for the continuous variables after verifying homogeneity of variances by Levene’s tests, and Chi-Square tests for the categorical variables (sex and laterality). Blinding integrity was assessed by a Chi-Square test. The significance level was set at p < 0.05, two-tailed for all analyses.

### fMRI data analysis

The fMRI data were analyzed with SPM12 software (Wellcome Department of Cognitive Neurology, London, UK). The first five volumes of each run were discarded to allow for signal stabilization. Brain images were realigned, normalized into the MNI stereotaxic space and smoothed with an 8-mm Gaussian filter. The first level analysis was conducted on each scan separately using the general linear model with the three image types as predictors (smoking, neutral and animal) and six motion confound predictors (x, y, z translation and rotation). The [smoking-related – neutral] contrast was generated for each participant. We performed a one-sample t-test for this contrast, using the first (baseline) scan from each individual before entering the tDCS protocol. For the whole-brain analyses, a combined voxelwise threshold of p < 0.005 and a spatial extent threshold of 85 voxels was used to achieve an α of 0.05, corrected for multiple comparisons. The minimum cluster size required for corrected significance was determined with a Monte Carlo simulation of 1,000 iterations (Slotnick *et al*.^26^). This combination of intensity and extent thresholds has been considered appropriate in yielding a good balance between Type I and II error rates^27^.

Finally, to investigate the effect of tDCS on brain activity, the first-level contrast was entered in a flexible model with Time as a within-subject factor (2 levels: before and after the 10 sessions) and Stimulation as a between-subject factor (2 levels: active and sham). A Time by Stimulation F-test was generated to visualize the interaction, using a voxel height threshold at p < 0.005 significance and minimum k of 85 voxels. Corresponding brain regions were identified with the WFU PickAtlas implemented in SPM^28^. To investigate whether the significant clusters corresponded to increased or decreased activity, in a following step, post hoc paired t-tests were performed on beta values between pre and post tDCS sessions in active and sham groups at p < 0.05. As an exploratory analysis, the association between changes in brain activity and cigarette consumption was investigated using Pearson correlation test.

### Data availability

The datasets analyzed during the current study are available from the corresponding author on reasonable request.

## Results

### Participant’s characteristics at baseline

Twenty-nine participants with TUD were included in our analyses (17 in the active group, 12 in the sham group). Demographic and clinical measures are presented in Table 1. There were no statistical differences between active and sham groups at baseline for gender, age, handedness, mood, duration of smoking, motivation to quit, level of dependence, levels of CO and cigarette consumption reported at inclusion. tDCS was well-tolerated and no adverse events were reported. Blinding integrity analysis showed no difference between groups (Chi-square = 2.83; p = 0.24).

**Table 1:** Baseline demographic and clinical characteristics of participants enrolled in active and sham groups.

	Active Group (N = 17)		Sham Group (N = 12)		p-value*
	Mean	SD	Mean	SD	
Gender (Male/Female)	5/12		4/8		0.82
Handedness (Right/Left & Ambidextrous)	12/5		11/1		0.36
Age (years)	41.2	9.1	40.8	9.4	0.90
Duration of smoking (years)	21.4	7.4	22.9	7.9	0.68
Q-MAT score (motivation to quit)	14.9	3.6	15.5	2.9	0.66
FTND score (level of dependence)	6.5	1.0	6.8	1.6	0.68
Cigarette consumption reported at inclusion	17.6	4.4	20.6	3.7	0.07
Beck Depression Inventory score (Mood)	2.0	2.4	2.2	2.6	0.86
Level of CO in exhaled breath (ppm)	13.3	9.4	14.3	8.1	0.78

CO, Carbon Monoxide; FTND, Fagerstrom Test for Nicotine Dependence; N, Number; Q-MAT, “Questionnaire de Motivation à l’Arrêt du Tabac”; SD, Standard Deviation.

*Student’s t and chi-square tests were conducted to assess group differences for continuous and categorical variables, respectively.

### Effects on smoking intake

**Figure 1:**
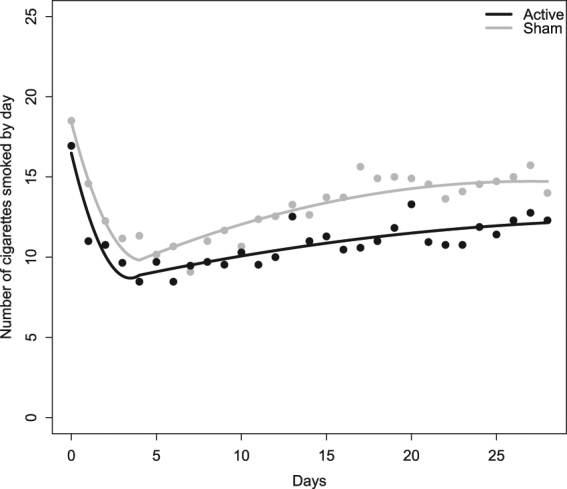
Changes in cigarette consumption over time in both stimulation group (active, N = 17, and sham, N = 12). Cigarette consumption consisted in the day-by-day reported number of cigarettes smoked. The lines show the predicted values from the linear mixed-effects model. Dots show means from the data. Active stimulation is represented in black and sham stimulation is represented in grey. Values at Day 0 represent the baseline cigarette intake the day before the beginning of stimulation. Values at Day 1 to Day 5 represent the cigarette intake during the stimulation’s course (2 sessions by day). Values at Day 6 to Day 28 during the follow-up assessment. The figure has been plotted using R software [v.3.4.3] (R Core Team^25^. R: A Language and Environment for Statistical Computing, R Foundation for Statistical Computing, Vienna, Austria. https://www.R-project.org).

Among the 29 participants who completed the study, one presented with missing data for smoking consumption from day 10 to 28 (in the sham group). Results from the linear mixed-effects model (Fig. 1) showed a significant decrease in the number of cigarettes smoked during tDCS treatment (linear time effect p < 0.001, quadratic time effect p = 0.006) and a significant increase in cigarettes smoked during the follow-up period (linear time effect p < 0.001, quadratic time effect p = 0.005). There were no significant differences between groups regarding the number of cigarettes smoked at baseline (p = 0.215), the decrease during treatment (linear effect p = 0.708, quadratic effect p = 0.480) or the increase during follow-up (linear effect p = 0.891, quadratic effect p = 0.494). A spaghetti plot of individual trajectories for the number of cigarettes smoked in both groups and details of the estimates of the model are provided in Supplementary Material S4 and S5.

Regarding CO levels, five participants presented with missing data at one-month, analyses were thus computed on 24 participants (13 in the active group and 11 in the sham group). The ANOVA revealed a significant main effect of Time (F = 7.46; p = 0.002; η^2^ = 0.253) but no effect of Group (F < 0.01; p = 0.983; η^2^ < 0.001) and no interaction between Time and Group (F = 0.14; p = 0.868; η^2^ = 0.006).

### Effects on smoking craving

**Figure 2:**
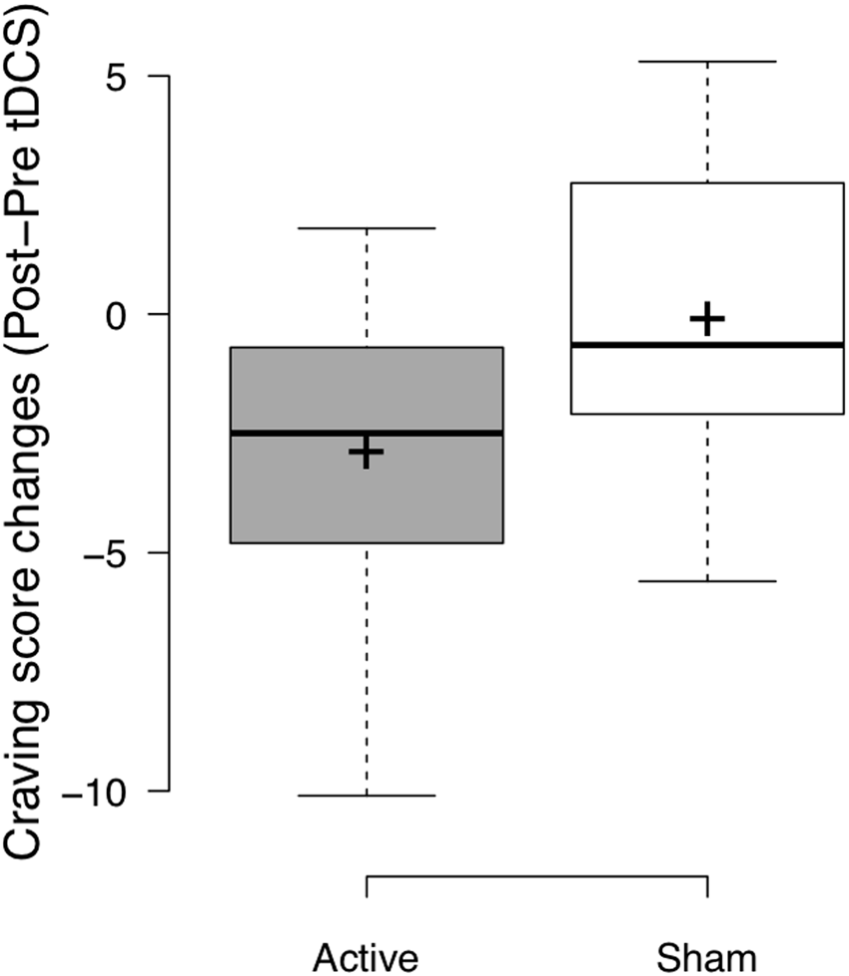
Box plots of the craving scores changes (Post-Pre) following transcranial Direct Current Stimulation (tDCS) in each stimulation group (active, N = 17, and sham, N = 12) regardless of the tDCS session. Active stimulation is represented in dark grey and sham stimulation is represented in white. Center lines show the medians; box limits indicate the 25th and 75th percentiles as determined by R software; whiskers extend 1.5 times the interquartile range from the 25th and 75th percentiles, crosses represent sample means. The figure has been plotted using R software [v.3.4.3] (R Core Team^25^. R: A Language and Environment for Statistical Computing, R Foundation for Statistical Computing, Vienna, Austria. https://www.R-project.org).

Regarding changes in craving scores from pre to post tDCS (Post-Pre tDCS), the ANOVA revealed a significant main effect of Session (F = 3.057; p = 0.002, η^2^ = 0.102), the first tDCS session showing a significantly different profile than other tDCS sessions. Moreover, a significant main effect of Group (F = 5.162; p = 0.031; η^2^ = 0.160; Fig. 2) was reported. Participants receiving active tDCS displayed significant higher reduction in craving scores after tDCS than participants receiving sham tDCS. No interaction between Session and Group (F = 1.265; p = 0.257; η^2^ = 0.045) was reported.

### Baseline scan – Event-related cue-exposure paradigm

**Figure 3:**
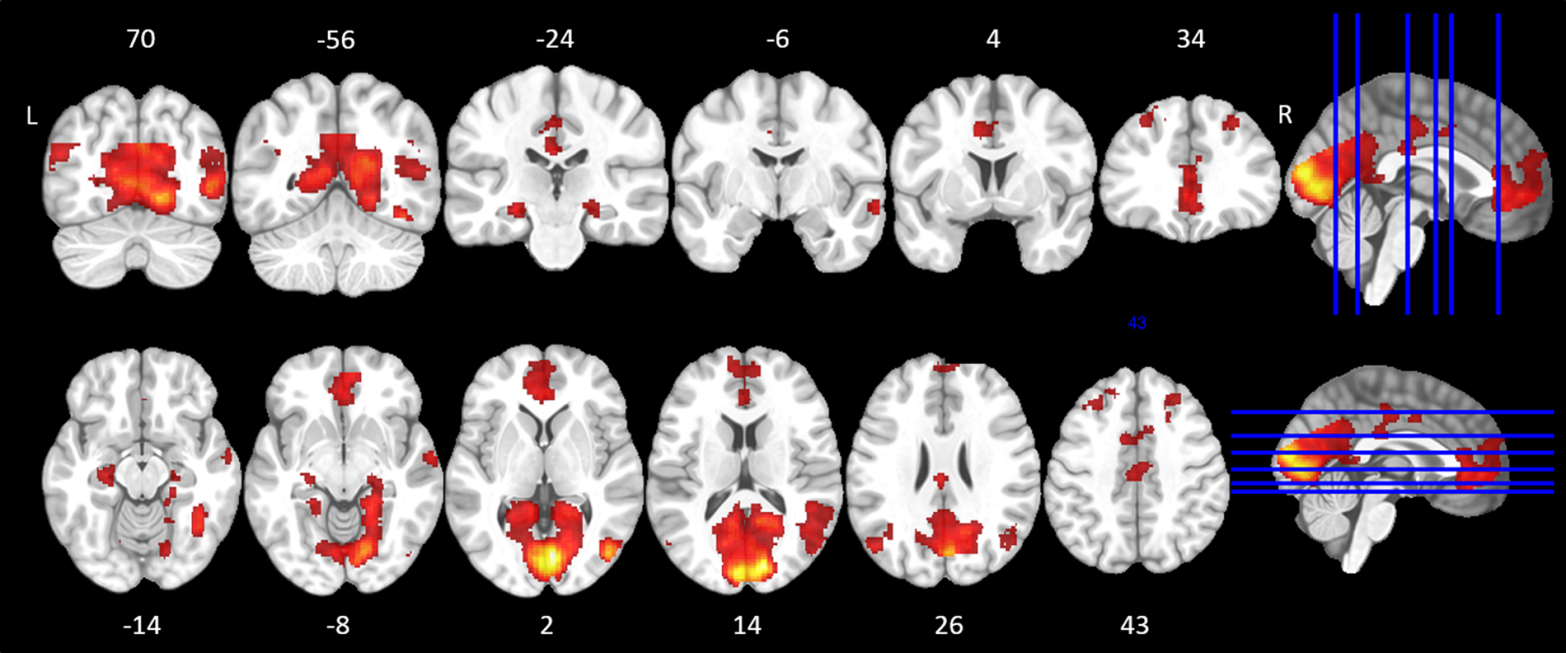
Axial and coronal views of brain reactivity to smoking vs. neutral cues in adults with tobacco-use disorder (N = 24). Significant clusters were overlaid on an anatomical MNI template. These analyses were performed at a voxel height threshold at P < 0.005 significance (minimum K = 85).

Imaging data from 24 subjects were analyzed (14 in the active group and 10 in the sham group). The smoking minus neutral contrast at baseline yielded eleven significant clusters, including the extended visual system (bilateral cuneus and lingual gyrus, right fusiform gyrus), the right middle and superior temporal gyrus, the left angular gyrus, the cingulate gyrus (anterior and posterior), the left hippocampus, the medial frontal gyrus and bilateral superior frontal gyri (Table 2 and Fig. 3).

**Table 2:** Results of the one-sample t test for the smoking minus neutral contrast at baseline.

Brain region	BA	Cluster level		Voxel level			Peak MNI coordinates		
		size	p	T	Z	p (unc)	x	y	z
R and L Cuneus/Lingual gyrus	30/18	9488	<0.001	13.46	7.02	<0.001	6	−78	2
				12.64	6.84	<0.001	12	−86	12
				11.14	6.47	<0.001	−4	−92	10
R Middle/Superior temporal gyrus	39/13	1351	<0.001	8.68	5.73	<0.001	46	−76	2
				4.86	3.99	<0.001	40	−60	20
				4.70	3.89	<0.001	48	−44	14
R Fusiform gyrus	37	195	0.005	6.20	4.71	<0.001	42	−56	−16
Medial frontal gyrus/Anterior cingulate	10/32	1956	<0.001	5.93	4.58	<0.001	4	54	−4
				5.70	4.45	<0.001	−2	38	0
				5.48	4.34	<0.001	0	40	−12
L Hippocampus/Parahippocampal gyrus		151	0.011	4.98	4.06	<0.001	−26	−24	−12
				3.68	3.23	0.001	−22	−16	−16
L Angular gyrus/Middle temporal gyrus	39	311	0.001	4.81	3.96	<0.001	−50	−70	24
				3.71	3.25	0.001	−42	−64	32
				3.57	3.15	0.001	−40	−62	24
Cingulate gyrus	24	292	0.001	4.68	3.88	<0.001	−2	−24	26
				4.19	3.57	<0.001	−2	−20	42
				3.86	3.35	<0.001	4	−14	40
R Middle temporal gyrus	21	107	0.028	4.62	3.85	<0.001	64	−6	−8
Cingulate gyrus	24/32	145	0.012	4.46	3.75	<0.001	−10	4	40
				3.62	3.18	0.001	0	0	38
				3.21	2.89	0.002	10	12	42
L Middle/Superior frontal gyrus	8/9	339	<0.001	4.05	3.48	<0.001	−26	26	32
				4.04	3.48	<0.001	−18	26	58
				3.66	3.21	0.001	−30	30	44
R Middle/Superior frontal gyrus	8/9	136	0.015	4.00	3.45	<0.001	26	34	44
				3.43	3.05	0.001	22	18	42

p = 0.005 uncorrected; k = 85; BA = Brodmann area; L = Left; R = Right.

### Effects on brain reactivity to smoking cues measured by fMRI

**Figure 4:**
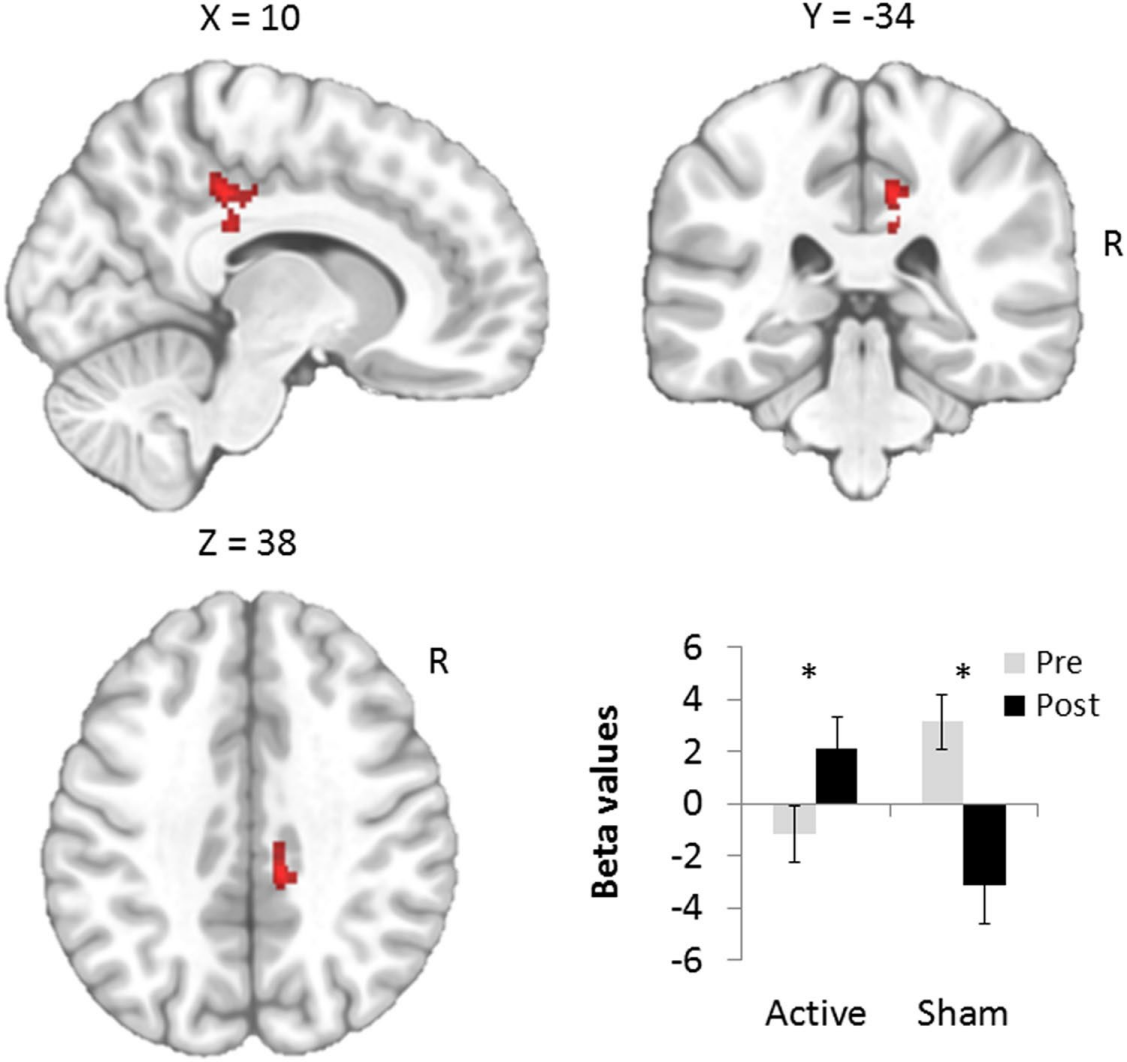
Sagittal, coronal and axial views of significant Group x Time interaction for the smoking versus neutral cues contrast. These analyses were performed at a voxel height threshold at p < 0.005 significance (minimum K = 85). The significant cluster was overlaid on an anatomical MNI template. The diagram depicts beta values extracted from the right posterior cingulate cluster for each time (pre and post) and group (active, N = 14 and sham, N = 10).

The interaction between Time and Stimulation produced one significant cluster in the right dorsal posterior cingulate cortex (PCC; BA31/BA23; Table 3 and Fig. 4). Post hoc paired t-tests on beta values showed that 10 sessions of active tDCS increased activity in the right PCC during the cue-reactivity paradigm (T = −2.713; p = 0.018) while 10 sessions of sham tDCS decreased PCC activity (T = 3.148; p = 0.012). Exploratory analyses showed no significant correlation between the changes in PCC brain reactivity and changes in cigarette consumption at endpoint (r = −0.046, p = 0.831) and between the PCC brain reactivity at baseline and the number of cigarette smoked at endpoint (r = −0.210, p = 0.326) in the whole sample (n = 24).

**Table 3:** Results of the flexible factorial model for the smoking > neutral contrast at baseline vs after the 10 sessions of transcranial direct current stimulation in the active (N = 14) and sham groups (N = 10).

Brain region	BA	Cluster level		Voxel level			Peak MNI coordinates		
		size	p	F	Z	p(unc)	x	y	z
R Posterior cingulate gyrus	31/23	96	0.019	16.63	3.56	<0.001	10	−34	38
				13.68	3.24	0.001	10	−32	28
				10.92	2.90	0.002	8	−26	38

p = 0.005 uncorrected; k = 85; BA = Brodmann area; R = Right.

## Discussion

To our knowledge, this is the first sham-controlled study that evaluates the effects of 10 repeated sessions of tDCS on smoking, craving and brain activity in adults with TUD with a one-month follow-up. We found that 10 sessions of active tDCS significantly reduced smoking craving and increased brain reactivity to smoking-cues within the right PCC as compared to sham but that both active and sham tDCS significantly reduced cigarette consumption for at least one month.

The lack of differential effects between active and sham tDCS on cigarette consumption is contrary to our hypothesis and some previous studies measuring cigarette smoking^11,13^. It may be due to several factors, such as study design, stimulation parameters, and studied population.

First, the sham effect reported in our study might be explained by the protocol design: participants were instructed to refrain from smoking 90 min before each tDCS session, two times a day. This instruction led to a 4-hour abstinence period each day (90 min prior to each session added to 30 min of visit), which may have reduced cigarette consumption in both groups during the 5 days of tDCS. Moreover, the use of a parallel design may have contributed to a stronger placebo effect as compared to previous studies using a crossover design^13^. Indeed, a recent meta-analysis investigated the impact of the study design on the placebo effect in noninvasive brain stimulation studies, revealing a significant effect of placebo in parallel studies but not in crossover studies^29^. Our study is limited by the fact that the sham group unfortunately had more drop out. However the link with the sham treatment is unlikely since participants dropped out before receiving any tDCS session. In addition, even though our study had the longest follow-up period among tDCS protocols for smoking cessation, one month may be too short to overcome acute placebo effects. Another explanation that may have contributed to the lack of significant findings for smoking consumption is that primary outcome is prone to a bias of underreporting. The inclusion of other outcomes such as latency to smoke or the total number of cigarettes smoked in one hour following the tDCS session would have been relevant, as done in Falcone *et al*. study^12^. However, our study also included a more objective measure of smoking consumption, CO levels, and found no significant difference between active and sham tDCS.

Secondly, regarding stimulation parameters, our electrode montage consisted in placing the anode over the right DLFPC and a large cathode over the left occipital regions whereas prior studies showing an effect of repeated sessions of tDCS on cigarette consumption have used a bifrontal tDCS montage. In Boggio *et al*.^11^, the anode was placed over F3 and a large cathode over F4 and in Fecteau *et al*.^13^ the anode was placed over F4 and the cathode over F3. Interestingly, computational modeling of tDCS electrode montages has shown that the brain electrical field distribution varies according to the size and position of electrodes^30,31^. Montages with wider spacing between electrodes resulted in less shunting and more current entering the brain but less electrical field levels in the DLPFC. In addition, while bifrontal montages have been shown to result in strong stimulation of both DLPFC, fronto-occipital montages resulted in greater activation in deeper brain structures such as the anterior cingulate cortex. Here, estimation of the electric field distribution for the tDCS montage used (anode over the right dorsolateral prefrontal cortex and cathode over the left occipital areas, Supplementary Fig. S3) showed a widespread electric field distribution in both hemispheres including the right frontal and precentral areas, the cingulate, and the left postcentral and temporal areas. Further studies are needed to establish the optimal electrode montage in subjects with TUD. The number and frequency of repeated tDCS sessions might also have an impact on smoking outcomes. Previous studies reporting positive findings on smoking have delivered 5 daily sessions^11,13^. Delivering 10 sessions of tDCS twice daily might have lesser effects on smoking but the impact of the number of tDCS sessions is not yet documented.

Some participants’ characteristics may have contributed to differential effects of tDCS. In our study, participants were adults diagnosed with TUD with a mean age of 41.0 (SD = 9.1) who had high motivation to quit smoking as assessed by the Q-MAT. Participants smoked an average of 18.9 (4.3) cigarettes by day for an average of 22.2 (7.4) years and their dependence was rated as high (6.6 (1.3)) according to the FTND. Thus, our population differed from previous studies showing an effect of tDCS on cigarette consumption, which recruited younger participants who smoked an average of 15 cigarettes by day and showed a low to moderate dependence (mean FTND scores of 4.4 and 5.7 respectively^11,13^). Interestingly, it has been observed that a higher level of dependence is associated with less success in smoking cessation^32^ and that older age is associated with less response to brain stimulation in patients with psychiatric conditions^33^. Moreover, since we recruited participants that showed high motivation in quit smoking, another explanation of the lack of significant findings is the pure placebo response that could have mobilized the participants to pursue concomitant strategies to decrease smoking. However, to reduce this possibility, we specifically asked participants to not use nicotine-replacement strategies or medications for smoking-cessation during the protocol time course.

Finally, participants’ smoking status at the moment of the stimulation may have influenced tDCS outcomes. Of note, in our study, participants were not explicitly instructed to stop smoking during the protocol but we controlled time between the last cigarette smoked and stimulation by asking participants not to smoke 90 minutes before each tDCS session. By asking participants not to smoke 90 minutes before each tDCS session, we would like to limit the occurrence of an interaction between stimulation and nicotine aftereffects that might have limited tDCS effects. Indeed, several studies reported an alteration of tDCS-induced neuroplasticity following nicotine or nicotinic receptor partial agonist administration^34–36^. However, by doing so, tDCS was performed during nicotine withdrawal and it may also have limited the effects of tDCS. Indeed, a study reported that tDCS-induced neuroplasticity was abolished by nicotine withdrawal but restituted by nicotine administration^37^. Accordingly and since tDCS has shown potential to increase ability to resist smoking after an overnight abstinence^12^, the effects of tDCS on smoking might be improved by combining with nicotine administration in abstinent participants^38^. Further studies are needed to better investigate the interaction between nicotine and tDCS effects and to find the optimal strategy for the use of tDCS in TUD.

Although no differences were reported between active and sham tDCS on cigarette consumption, we did report a significant different effect of active and sham tDCS on smoking craving. Namely, a diminution of craving was observed after active tDCS sessions as compared to sham but we did not observe a cumulative effect of sessions on craving diminution. The reduction of smoking craving observed after tDCS is consistent with studies showing that tDCS reduced cue-induced craving^10,11,13,39^ and background/tonic smoking craving^10^. The distinct effects of tDCS on smoking and craving suggested distinct brain underpinnings.

Moreover, we investigated brain reactivity to smoking cues before and after the 10 sessions of tDCS with event-related fMRI. When comparing the smoking versus neutral cues, the event-related cue-exposure at baseline elicited brain activity changes in the extended bilateral visual system, the right middle and superior temporal gyrus, the left angular gyrus, the anterior and posterior cingulate gyri, the hippocampi, the medial frontal gyrus and bilateral superior frontal gyri. The involvement of these brain areas in craving are in line with recent meta-analyses of neuroimaging studies of cue reactivity in TUD^8,16^. Interestingly, we found greater activity in bilateral superior frontal gyri corresponding to both DLPFCs during exposure to smoking cues, which sustains the choice of the DLPFC as a brain target for noninvasive brain stimulation in TUD. Our study highlighted that 10 sessions of active tDCS modulated brain reactivity to smoking cues as compared to sham. Namely, an increase in brain reactivity to smoking cues was observed within the right PCC after 10 sessions of active tDCS whereas a decrease in the right PCC was observed after 10 sessions of sham tDCS. The involvement of the PCC in TUD is not unexpected since it is one of the regions that show the largest brain responses to smoking cues^8^. Furthermore, the PCC has been linked to smoking craving^40^, attentional bias towards smoking cues^41^, but also cigarette smoking^42^ and abstinence^43^. Remarkably, the role of the PCC in resisting craving to smoke has been highlighted in a fMRI study comparing resisting craving and craving conditions during a cue-exposure fMRI task^44^. The authors observed higher brain activity within the PCC when subjects were actively trying to resist craving to smoke than when they allowed themselves to crave. Since we did not find any association between changes in PCC brain reactivity and cigarette consumption, we hypothesized that the significant increase in brain activity within the PCC after active tDCS is not related to cigarette consumption but might instead reflect a resist-to-crave strategy that is consistent with the observed decrease in craving.

In summary, we reported that 10 sessions of tDCS applied over the right DLPFC reduce craving in subjects with TUD and modulate activity within the right PCC, a key structure involved in intrinsic control networks and in the resisting craving to smoke network. However, these beneficial cognitive and brain modulations were not sufficient to lead to a significantly higher decrease of cigarette consumption as compared to sham.

## Electronic supplementary material

Supplementary Material
