# Supplementary material for: Effects of repeated transcranial direct current stimulation on smoking, craving and brain reactivity to smoking cues
Source: Sci Rep. 2018 Jun 7;8:8724. doi: 10.1038/s41598-018-27057-1 (PMC5992174; doi:10.1038/s41598-018-27057-1)
Supplement: Supplementary file 1 — Supplementary Material [file 41598_2018_27057_MOESM1_ESM.pdf]

Supplementary Material

Effects of repeated transcranial direct current stimulation on smoking, craving and brain reactivity to smoking cues

Marine Mondino [1,2,3,*], David Luck [4], Stéphanie Grot [4,5], Dominique Januel [6], Marie-Françoise Suaud-Chagny [1,2], Emmanuel Poulet [1,2,3], Jérôme Brunelin [1,2,3]

Supplementary Figure S1. Time course of the study.

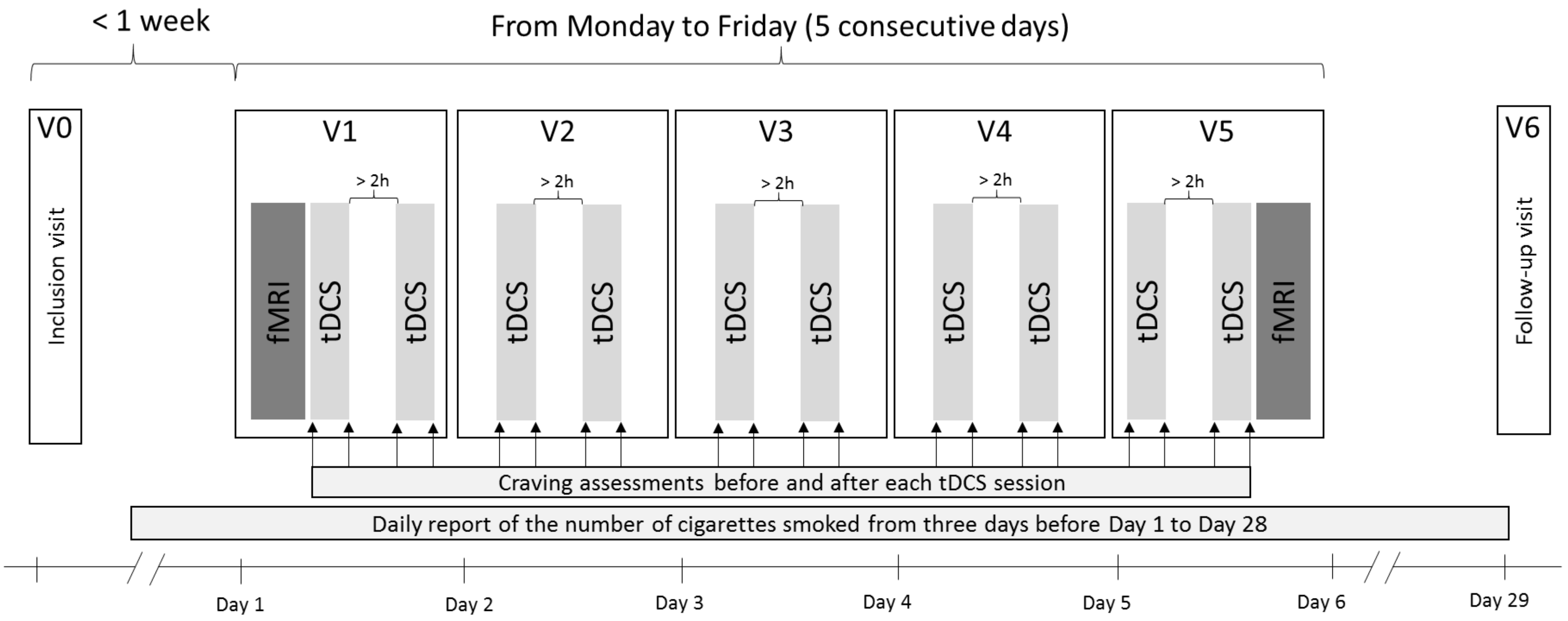

Supplementary Figure S2. Flowchart of the study.

CONSORT
TRANSPARENT REPORTING of TRIALS

CONSORT 2010 Flow Diagram

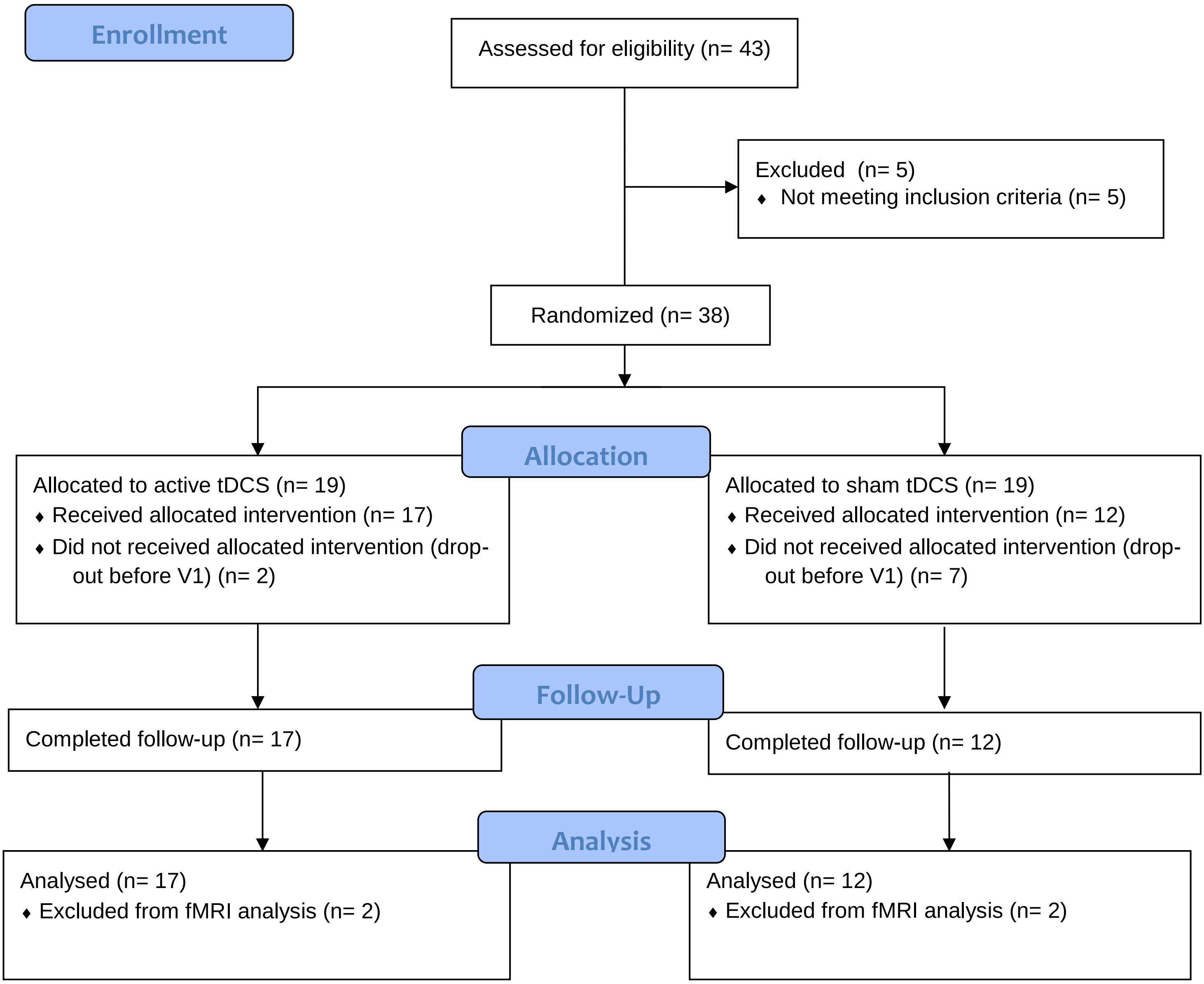

Supplementary Figure S3. tDCS electric field distribution estimate.

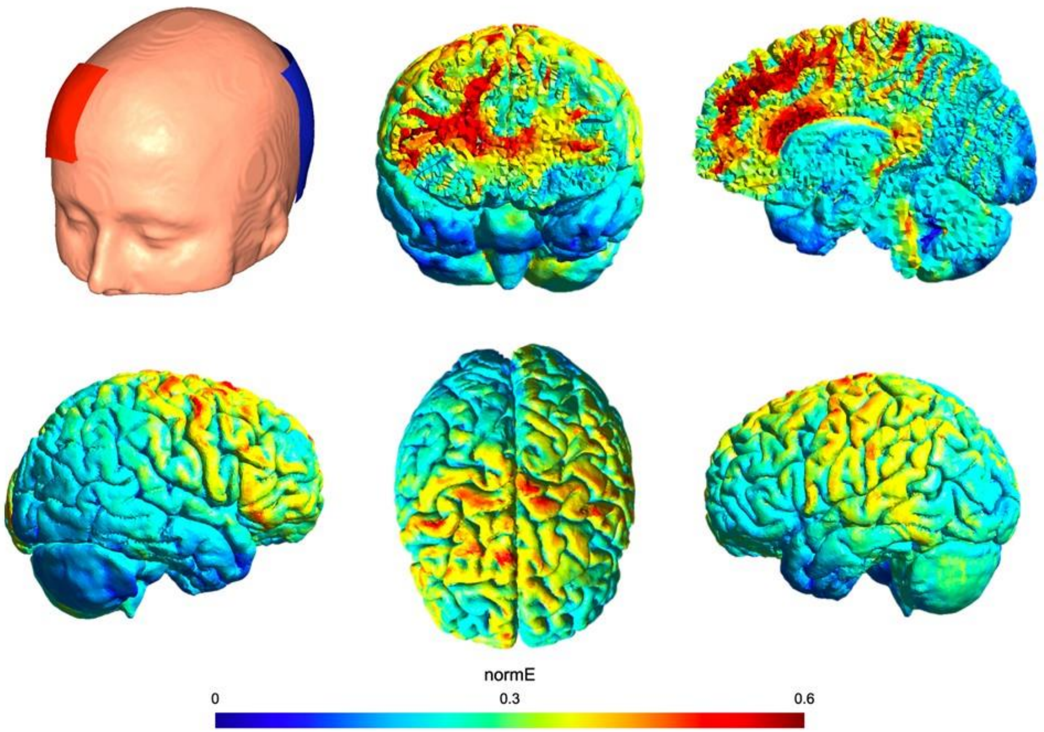

Results of the electric field distribution estimate for the tDCS montage with the anode over the right dorsolateral prefrontal cortex (Fp2-F4) and the cathode over the left occipital areas (O1-T5). The electric field strength is scaled from 0 (minimum: blue) to 0.6 mV/mm (maximum: red). Electric field simulation was performed with SimNIBS 2.0.1 using the standard head model provided by the software and a current intensity of 2 mA.

Supplementary Figure S4. Individual trajectories of the reported number of cigarettes smoked over time in active (N = 17, represented in black) and sham groups (N = 12, represented in grey). tDCS sessions were delivered from Day 1 to Day 5 (2 sessions per day).

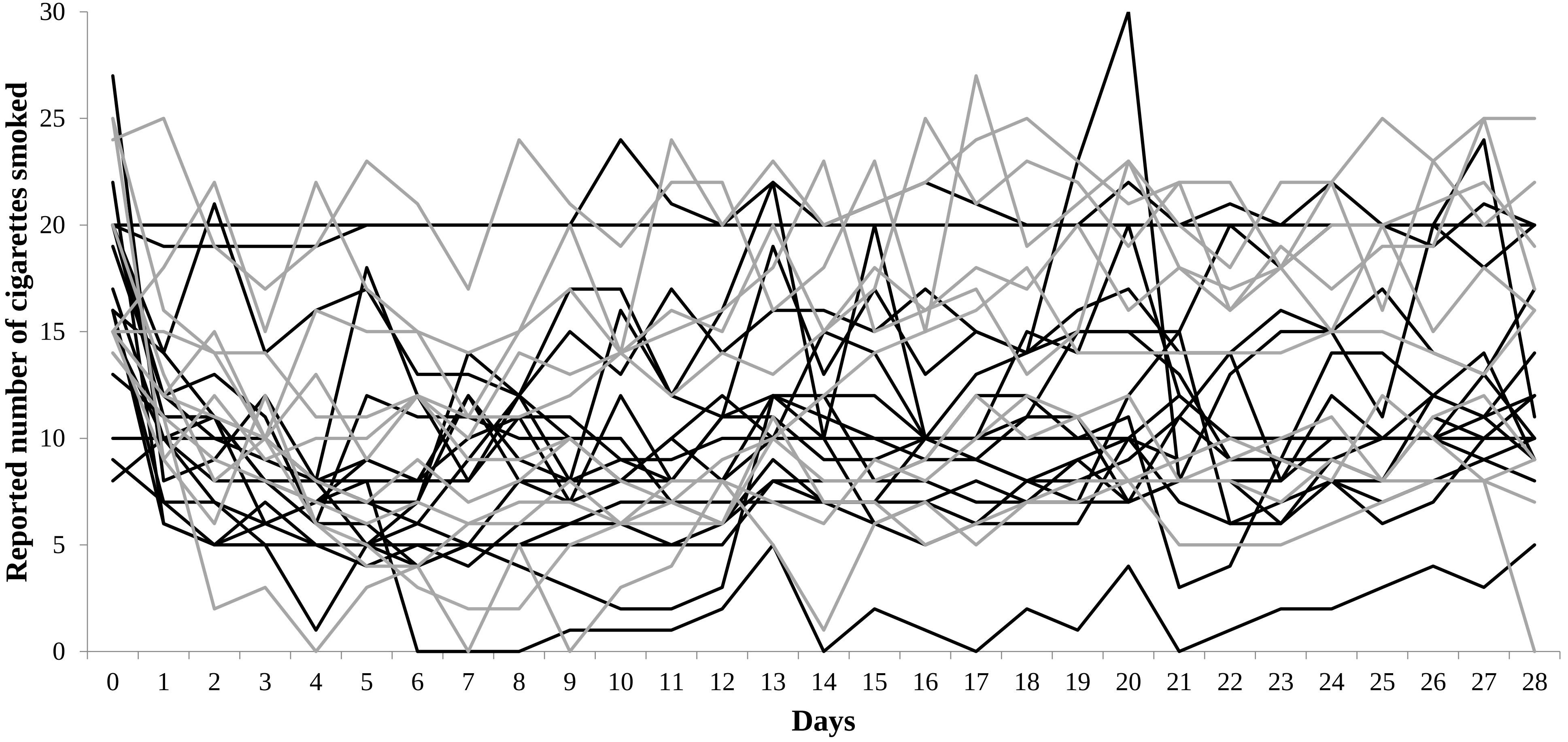

Supplementary Material S5. Parameter estimates from the linear mixed-effects model on the reported number of cigarettes smoked per day.

**Parameters**	**Estimates (β)**	**Standard Error**	**p**
(Intercept)	18.4	1.16	<.001
Group	-1.92	1.52	.215
Time1	-4.10	0.82	<.001
Quadratic Time1	0.49	0.18	.006
Time2	4.59	0.82	<.001
Quadratic Time2	-0.49	0.17	.005
Group x Time1	-0.40	1.07	.708
Group x Quadratic Time1	0.16	0.23	.480
Group x Time2	0.15	1.07	.891
Group x Quadratic Time2	-0.16	0.23	.494

Time1 represents the period during tDCS treatment and Time2 represents the follow-up period.
